# Heat inactivation of SARS-CoV 2 enabled the measurement of salivary cortisol during COVID-19 pandemic

**DOI:** 10.1007/s12020-023-03597-z

**Published:** 2023-11-22

**Authors:** Giacomo Voltan, Giorgia Antonelli, Alessandro Mondin, Irene Tizianel, Chiara Sabbadin, Mattia Barbot, Daniela Basso, Carla Scaroni, Filippo Ceccato

**Affiliations:** 1 Department of Medicine DIMED, University of Padova, Padova, Italy; 2 Endocrine Disease Unit, University-Hospital of Padova, Padova, Italy; 3 Laboratory Medicine Unit, University-Hospital of Padova, Padova, Italy

**Keywords:** Salivary cortisol, COVID-19, Sars-Cov2, Cushing, Pandemic, Cortisone

## Abstract

**Background and aim:**

Salivary cortisol has become an essential tool in the management of cortisol-related disease. In 2020 the sudden outbreak of COVID-19 pandemic caused several concerns about the use of saliva, due to the risk of contamination, and a European consensus further discourage using salivary cortisol. To decrease infectious risk, we handled specimens by applying a heat treatment to inactivate viral particles, further evaluating the impact of the COVID-19 pandemic on the use of salivary cortisol in clinical practice.

**Material and methods:**

Saliva samples were exposed for 10 min at 70 °C, then cortisol was measured using LC-MS/MS. The number of salivary cortisol examinations from 2013 to 2022 was extracted from the local electronic database: those performed in 2019, 2020, and 2021 were analyzed and compared with the historical data.

**Results:**

During 2020 we observed a decrease of 408 (−20%) examinations (*p* = 0.05) compared to 2019; especially in salivary cortisol daily rhythm and salivary cortisol/cortisone ratio (respectively reduction of 47% and 88%, *p* = 0.003 and *p* = 0.001). Analyzing year 2021 compared with 2020 we reported an increase of 420 examinations (+20%, *p* = 0.01), with a complete recovery of salivary cortisol measurement (considering 2019: *p* = 0.71). Major differences were observed between morning salivary cortisol (−20%, *p* = 0.017), LNSC (−21%, *p* = 0.012) and salivary cortisol rhythm (−22%, *p* = 0.056). No Sars-Cov2 infections related to working exposure were reported among laboratory’s employers.

**Conclusions:**

We speculate that the adoption of an appropriate technique to inactivate viral particles in saliva specimens allowed the safety maintenance of salivary collections, also during the Sars-CoV-2 outbreak.

## Introduction

The applications of salivary cortisol (F) in routine clinical practice include diagnosis and assessment of medical therapies, in either cortisol excess or deficiency settings.

The measurement of late-night salivary cortisol (LNSC) is a widely accepted, simple, reliable, non-invasive screening test for Cushing’s syndrome (CS). Periodic assessment of LNSC is also an excellent approach in monitoring post-surgical patients with Cushing’s disease in order to detect early relapses, even in the absence of clear clinical symptoms [1, 2]. Furthermore, in CS patients treated with medical therapy, there is increasing evidence regarding the use of LNSC as an additional tool to titrate therapy [3].

Adrenal insufficiency (AI) is a life-threatening condition, requiring long-life glucocorticoid (GC) replacement therapy [4]. Baseline morning unstimulated serum F levels are measured in patients with suspected AI, however, most of the commercially available F assays are not accurate enough in the low range of normality [5]. In recent years, measuring salivary F has been suggested for patients with AI [6], since it reflects serum-free F levels, and is not influenced by binding protein [7]. In addition, it is a non-invasive alternative to basal serum F levels [8, 9], as a parameter to increase the diagnostic accuracy in the corticotropin stimulation test in adult and pediatric patients [5, 10]. Recently, in the diagnostic work-up of AI, high diagnostic accuracy of salivary waking cortisone (E) has been reported, providing similar results to corticotropin stimulation test [11].

Currently, the most adopted modality for saliva samples’ collection is using the Salivette® (Salivette® Sarstedt, Numbrecht, Germany), which consists in an absorbent pad that is kept in the mouth for a standardized time (2–3 min) to soak up saliva [12–15]. One of the most reliable analytical methods to measure F and E in clinical practice is liquid chromatography coupled to tandem mass spectrometry (LC-MS/MS) [15].

In early 2020 the world experienced a sudden outbreak of COVID-19 infection, which was quickly re-classified as a pandemic by the World Health Organization. The clinical spectrum varied from asymptomatic carriers to patients with mild upper airway illness, up to those with severe progressive pneumonia multi-organ failure, and death [16].

Patients affected by endocrine chronic diseases were reported to be at high risk of a worse clinical outcome in case of COVID-19 infection. Moreover, before the release of vaccines, specific monoclonal antibodies and antivirals, COVID-19 treatment was essentially based on high-dose GC administration [17]. This might induce endocrine complications, such as adrenal insufficiency secondary to the GC withdrawal syndrome [18, 19].

In addition, public health restrictions were adopted in response to COVID-19, limiting access to clinics, so these patients faced many difficulties in terms of the management of their diseases. In such scenario, we adopted some strategic precautions: saliva samples could be safely collected at home, mailed to the laboratory for F or E measurement, and then a virtual consultation in telemedicine was held to further reduce the risk of infection [20]. Nonetheless, in early 2020 an European Society of Endocrinology (ESE) task force released a consensus about the management of Cushing’s syndrome during COVID-19. It has been suggested to avoid the use of salivary tests due to the potential viral contamination, unless appropriate measures to handle biohazard were adopted, to minimize the risk of contagion [21].

This limitation, however, might be cumbersome for physicians: salivary F assessment is an irreplaceable tool in monitoring patients with cortisol-related conditions.

In our center, we routinely use salivary F since 2006 (in LC-MS/MS since the end of 2013) and starting from May 2020, we adopted a method finalized to inactivate viral particles in saliva samples based on heat exposition (70 °C for 10 min) [22].

In our study, we aimed to evaluate the impact of the COVID-19 pandemic on the usage and safety of salivary cortisol; so, we retrospectively analyzed the number of procedures related to salivary cortisol.

## Materials and methods

### Saliva collection and pre-treatment

Samples were collected using Salivette® devices containing a cotton swab (Sarstedt, Nümbrecht, Germany) according to the manufacturer’s instruction. To ensure proper collection at home, written instructions were provided to patients. To prevent food or blood contamination, samples were collected at least 30 min before meals or liquid hiring. Every patient brushed their teeth at least 30 min before saliva collection and refrained from smoking or eating licorice on the day of collection. All samples were stored at 4 °C until delivered to the laboratory, where they were centrifuged at 2000 *g* for 10 min to remove particulate material. Until analysis the device (without swab) was stored at −20 °C, also to break down mucin. When frozen saliva samples were defrosted before analyses, Salivette® devices were left for 10 min at 70 °C in a heater. After this step, a second centrifugation was performed on each sample (10 min, 2000 g) to obtain a clear fluid (typical volume about 1 ml).

### Sample analysis

Salivary F and E levels were measured with a homemade LC-MS/MS method, accredited ISO15189:2012 from 2016 [23]. The whole description of the methodic and analytical process could be easily consulted in a previous paper from our group [14]. Briefly, a first off-line automated Solid Phase Extraction (SPE) was carried out, followed by an on-line SPE with chromatographic separation and detection by tandem mass spectrometry. A comparability test was performed on 41 patient saliva samples with standard procedure (SP) and with the newly developed heat inactivation addition (HI) to verify no change in salivary F and salivary E quantification with this step, as suggested by CLSI guidelines [24]. Passing Bablok regression was calculated to verify the agreement between the results, previously assessing linearity with Cusum test. Pearson correlation coefficient was used to measure the linear relationship. Statistical analyses were performed using MedCalc® (MedCalc Software Ltd, Belgium).

### Data collection

All data were extracted from the electronic database of the University Hospital of Padova. We considered every laboratory assessment related to salivary F and E from the late 2013s to 2021.

We further split all salivary F and E performances of years 2019, 2020, and 2021 in every month and we compared the means of those months. These performances included different types of examinations. 08.00 AM and 11.00 PM salivary F (corresponding to LNSC), 08.00 AM and 11.00 PM salivary F/E ratio, daily salivary F rhythm, further organized in six saliva samples in a day, daily salivary F/E rhythm, further organized in six saliva samples in a day, salivary F after low-dose (1 µg) short ACTH (synacthen) test, salivary F after standard-dose (250 µg) ACTH test.

We compared all data regarding the year 2020, the first year of the COVID pandemic, with the same periods of 2019 and 2021 calculating the difference in the number of performances and every single kind of examination. We further compared data regarding year 2019 and 2021.

The database was managed, and statistical analysis was performed by Microsoft Excel and SPSS 24 software package for Windows (2016, IBM-SPSS, Armonk, New York, USA). Data are reported as absolute and relative frequencies for categorical variables. Quantitative variables were compared through a student two-tail *t*-test. A *p* < 0,05 was considered statistically significant. All datasets are included in the repository database of the University of Padova [25].

The study was conducted using anonymized records. We collected an aggregate number of data, all managed with a standardized anonymization process that assigned a unique anonymous code to each patient, without any possibility of back retrieving the subject’s identity, therefore single patient consent was not collected.

## Results

**Fig. 1:**
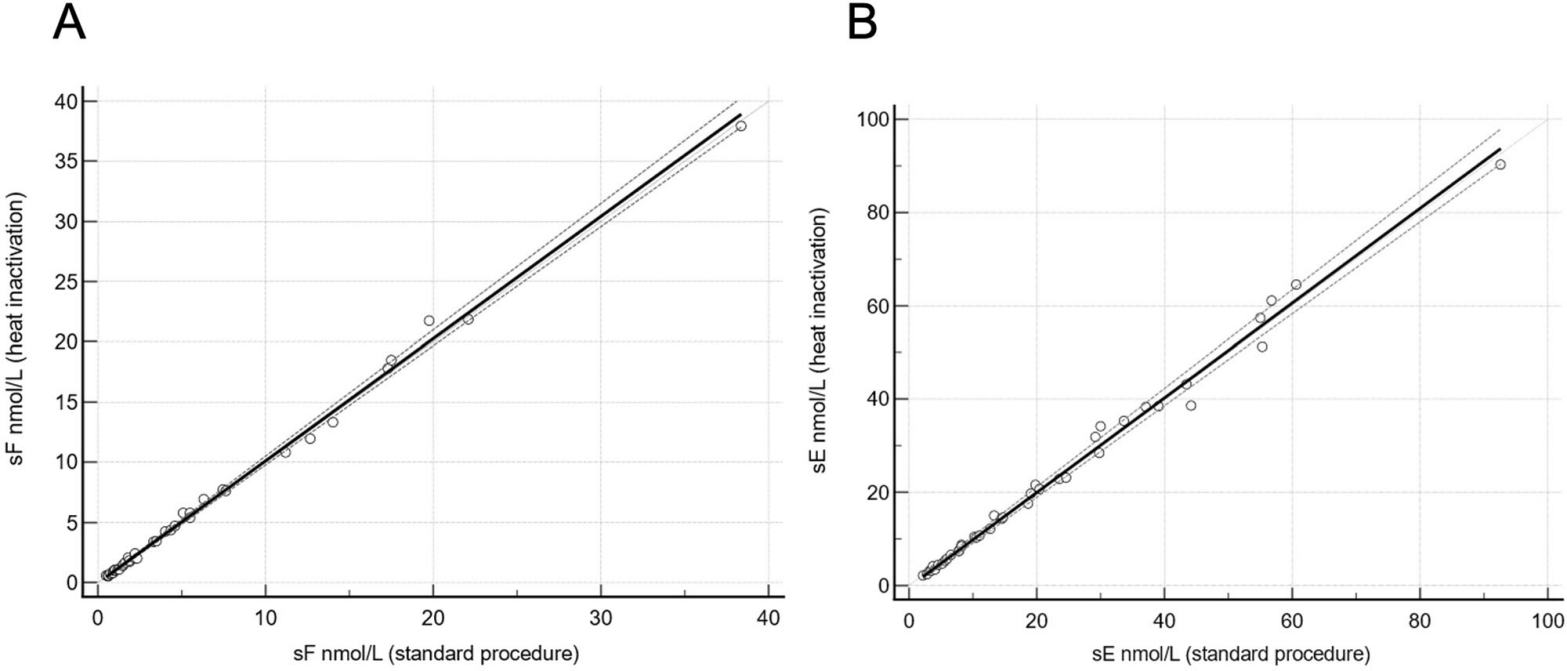
Scatter plot with Passing Bablok regression. Regression line (black) with confidence intervals (dotted) and line of best fit (gray) between standard procedure and heat inactivation addition for sF (**A**: y = 1.01x − 0.04) and for sE (**B**: y = 1.01x-0.33)

The Cusum linearity test showed a linear relationship between SP and HI (*p* > 0.1), for salivary F and salivary E. The regression between the procedures was [HI]=1.01 x [SP] −0.04, for salivary F and [HI]= 1.01 x[SP]− 0.33 for salivary E, with no significant bias considering the 95% confidence intervals for the proportional (0.99 to 1.05 and 0.98 to 1.06) and the constant bias (−0.01 to 0.01 and −0.75 to 0.03) (Fig. 1) and with a Pearson coefficient of 0.996 and 0.998, for salivary F and salivary E, respectively.

**Fig. 2:**
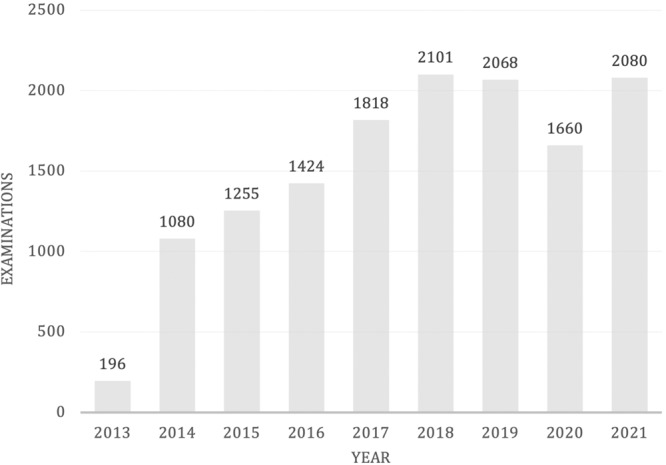
Total number of salivary cortisol assessments sorted by year from 2013

Since salivary F was adopted in our center, from October 2013, a total of 13.682 examinations had been performed until December 2021, with a progressive increasing trend during the years. However, as depicted in Fig. 2 the year 2020 was characterized by a drop in the salivary examinations due to COVID-19 pandemic.

Considering the year 2020 compared with 2019 we observed a decrease of 408 salivary samples (Δ-20%, *p* = 0.05). The major drop was observed in the first four-month (−32%) rather than in the second (−12%) and third four-month (−14%) of 2020.

Morning salivary F and LNSC decreased respectively by 73 (Δ−11%, *p* = 0.29) and 171 (Δ−16%, *p* = 0.12) tests, however, the most significant differences were detected analyzing daily salivary F rhythm and daily salivary F/E rhythm with a 47% (*p* = 0.003) and 88% (*p* = 0.001) reduction respectively. The total number of salivary F after the standard-dose (250 µg) ACTH test decreased by 67% (*p* = 0.26) while the amount of morning and bedtime salivary F/E ratio and salivary F after 1 µg ACTH test did not show a statistically significant difference, as shown in Table 1.

**Table 1:** Comparison of salivary cortisol (F) and cortisone (E) examinations between the year 2019 and 2020

Saliva Examination	2019	2020	Δ^2020-2019^ (n°)	Δ^2020-2019^ (%)	*p*
Morning salivary F	650	577	−73	−11%	0.29
Late night salivary F	1053	882	−171	−16%	0.12
Late Night F/E ratio	55	1	−54	−98%	0.00005
Morning F/E ratio	10	24	14	140%	0.058
F salivary rhythm	161	86	−75	−47%	0.003
F/E salivary rhythm	40	5	−35	−88%	0.001
250 µg ACTH test	15	5	−10	−67%	0.026
1 µg ACTH test	84	80	−4	−5%	0,85
Total	2068	1660	−408	−20%	0.05

**Fig. 3:**
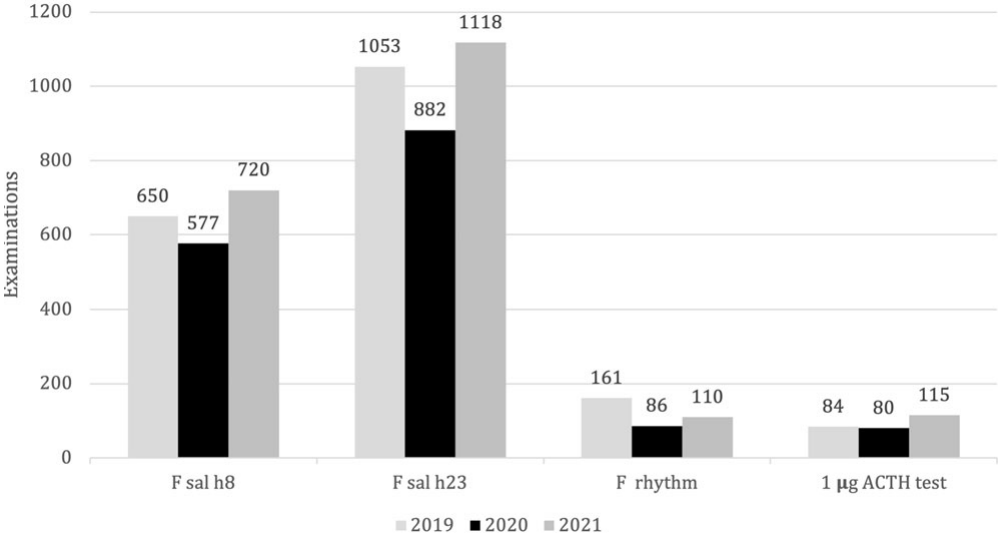
Distribution of salivary cortisol examination in the years 2019, 2020 and 2021

As pictured in Fig. 3 in 2021 the number of salivary F performances sharply increased after the reduction observed in the previous year. In 2020 were performed 420 examinations less than in 2021 (Δ = −20%) with a significant difference (*p* = 0.01); even in this case the major difference was noted in the first four-month of 2020 rather than the first one in 2021 (Δ = −30%).

In 2020, morning salivary F and LNSC were lower than in 2021 by 20% (*p* = 0.017) and 21% (*p* = 0.012) respectively. We rather observed other major differences between salivary daily F rhythm (−24, Δ = −22%, *p* = 0.056) and salivary F after 1 µg ACTH test (−35, Δ = −30%, *p* = 0.039). Conversely, the remaining performances show very few differences between the year examined, as reported in Table 2.

**Table 2:** Comparison of salivary cortisol (F) and cortisone (E) examinations between the year 2020 and 2021

Saliva Examination	2020	2021	Δ^2020,2021^(n°)	Δ^2020,2021^(%)	*p*
Morning salivary F	577	720	−143	−20%	**0.017**
Late night salivary F	882	1118	−236	−21%	**0.012**
Late night F/E ratio	1	0	+1	NA	NA
Morning F/E ratio	24	0	+24	NA	NA
F salivary rhythm	86	110	−24	−22%	0.056
F/E salivary rhythm	5	8	−3	−38%	0.39
250 µg ACTH test	5	9	−4	−44%	0.35
1 µg ACTH test	80	115	−35	−30%	**0.039**
Total	**1660**	**2080**	−420	−20%	**0.01**

Comparing data between the years 2019 and 2021 we observed similar results in terms of total performances, more precisely in 2019, 2068 tests were executed, while in 2021 the total amount was 2080 tests (Δ = −12, 1%, *p* = 0.71).

Considering the various kind of tests, morning salivary F and LNSC were similar in 2019 than in 2021 (respectively Δ = −10%, *p* = 0.16, Δ = −6%, *p* = 0.36); however, in both cases, there were no statistically significant results.

Daily salivary cortisol and salivary F after the 250 µg ACTH test were higher in 2019 too, respectively (Δ = +51, +46%, *p* = 0.067) and (Δ= + 6, +67%, *p* = 0.24) without reaching statistical significance (Table 3).

**Table 3:** Comparison of salivary cortisol (F) and cortisone (E) examinations between the year 2019 and 2021

Saliva Examination	2019	2021	Δ^2019–2021^(*n*°)	Δ^2019–2021^(%)	*p*
Morning salivary F	650	720	−70	−10%	0,16
Late night salivary F	1053	1118	−65	−6%	0,36
Late night F/E ratio	55	0	+55	NC	NC
Morning F/E ratio	10	0	+10	NC	NC
F salivary rhythm	161	110	+51	+46%	0,067
F/E salivary rhythm	40	8	+32	+400%	**0,003**
250 µg ACTH test	15	9	+6	+67%	0,24
1 µg ACTH test	84	115	−31	−27%	0,07
Total	**2068**	**2080**	−12	−1%	0,71

## Discussion

Salivary F is one of the cornerstones in the modern assessment of cortisol-related disease, especially in a referral center, where many patients with rare diseases are cured. There are several applications of salivary F, for example in the diagnosis of Cushing’s syndrome [26], but also in the follow-up of patients with Cushing’s disease, to detect relapse after surgical remission and while on medical therapy [1]. Over the last years, growing interest has been raised about the use of salivary F in patients with AI, either for diagnosis or to titrate glucocorticoid replacement therapy [7, 27]. We recently proposed new cut-offs for basal and stimulated salivary F to predict an intact HPA axis and rule out AI, especially in those patients with uncertain serum F values [28]. Moreover, the assessment of endogenous daily cortisol secretion computed with the area under the curve (AUC) can reveal over-treatment, even without clinical signs of hypercortisolism [29]. Therefore, in our referral center, as in many other centers of excellence, the role of salivary cortisol evaluation is fundamental in clinical practice.

In 2020, the COVID-19 pandemic determined an important impact on the national health system, due to the high number of patients with severe complications requiring hospitalization. On the other hand, most patients with Sars-Cov2 infections were asymptomatic or presenting mild symptoms, thereby, they could potentially transmit the virus to healthy people [30]. For all these reasons, the whole National Health System put a lot of effort to re-organize hospitals and outpatients’ clinics. Patients’ attendance was reduced, and unnecessary visits were delayed, in order to minimize the risk of infections.

Our Endocrinologic Unit normally takes care of a multitude of patients with chronic endocrine diseases. Obviously, follow-up visits and endocrinological examinations are of outmost importance to treat patients and to reduce and prevent comorbidities. Hence, it’s easy to understand how all these restrictive measures could have negatively affected our clinical practice.

Sars-Cov2 transmission is essentially mediated by the droplets of affected subjects [30]. Saliva was immediately and universally recognized as a potential vector for virus transmission. Indeed, the high viral load in this fluid has been successfully used to validate saliva specimens as a diagnostic test for Sars-Cov2 infections [31].

Therefore, the sudden outbreak of COVID-19 in the early 2020 s caught us unprepared, particularly in handling saliva specimens that were routinely collected for salivary cortisol assays. Moreover, in order to propose a conservative approach, in early 2020 an ESE task force suggested to avoid the use of salivary tests, due to the potential viral contamination [21]. This was suggested unless the local laboratories have instituted adequate measures to handle the specimens. However, no mentions of which measures might be the most appropriate were reported.

In our study, we demonstrated a significant drop in all salivary cortisol examinations comparing 2020 to 2019 (Δ-20%). Examinations whose numbers particularly decreased were those with multiple daily saliva sample collections, like daily salivary F rhythm and F/E ratio daily rhythm. Those numbers in 2020 were respectively lower by 47% and 88% than in 2019, and by 22% and 38% than in 2021. Their application was replaced by morning salivary F and LNSC, despite a different clinical significance.

In our referral center, we have been using salivary steroid assay since 2006 first with a radioimmunoassay [8], then, since 2013, moving to liquid chromatography coupled with tandem-mass spectrometry method [14]. Hence, we progressively acquired a well-established experience in using salivary steroids and we firmly believe in its importance and essential role, during the diagnosis and follow-up of patients with cortisol-related diseases. Consequently, the loss of these irreplaceable examinations would have been deleterious for both physicians and patients.

According to these reasons, from May 2020 our laboratory developed a novel precise protocol finalized to decrease the risk of contamination of health operators. Indeed, all saliva specimens were heated to 70 °C for 10 min to inactivate viral particles. Comparability tests with unheated specimens were performed, verifying no change in steroids quantification (Fig. 1).

Considering this assumption, we applied this method to all saliva samples collected during the COVID-19 pandemic, trying to minimize the decrease in salivary F and E examinations.

In addition, patients also had the possibility to collect the specimens at home, storing it in the fridge, and sending them to our center.

The efficacy of the heat in inactivating viral particles of Sars-Cov2 has been recently proved by several studies [32, 33], where the temperature and time of heat’s exposition were similar to our protocol. Different methods which revealed effectiveness in inactivating viral particles of Sars-Cov2 were based on UltraViolet-C usage [34–37]. Analyzing the role of chemical inactivation of Sars-Cov 2, buffers containing guanidine thiocyanate have proved to be useful for the isolation of viral nucleic acids and PCR-based analysis. Widera et al. tested several buffers containing guanidine isothiocyanate showing that most of them were able to completely inactivate samples containing SARS- CoV-2 [38]. The same authors evaluated that other chemical disinfectants and fixation solutions (acetone/methanol, ethanol, and paraformaldehyde) were suitable to completely inactivate the virus. Similar methods have been reported also in other papers: they can be included in routine clinical practice if they do not affect on work and reporting times [39–41].

Keeping in mind that heat inactivation was available from May 2020, the drop in salivary F and E examinations observed in 2020 than the previous year (*p* = 0.05), especially in the first fourth-month, is also probably related to the necessary time to develop the method.

Moreover, when the ESE consensus [21] was released, in July 2020, our center had already adopted heat inactivation. This is the confirmation that the close collaboration between endocrinologists and laboratory technicians was able to provide a quick and effective response to safely manage saliva-related examinations.

Therefore, our study showed a significant increase in salivary F examinations during the whole 2021 compared to 2020 (*p* = 0.01). Interestingly, data regarding 2021 are similar to those observed in 2019 (*p* = 0.71) in terms of the total amount of salivary F examinations.

The importance of these numbers should be evaluated considering the contextual trend of contagions in Italy during 2021, when the population experienced the third and fourth waves of COVID-19. Indeed, a total of 4.237.257 cases of contagions were detected in Italy, while in Veneto, our region, the cases detected were 396.339. On the other hand, during 2020 the number of contagions in Italy was 2.169.116, while in Veneto region it was 264.816 [42]. So, if we analyze these data, it’s easy to understand how in 2021 COVID-19 pandemic was far from a conclusion.

We have to acknowledge some limitations, first the retrospective design and then the “clinical” bias of our effort to continue to use salivary determinations, in partial disagreement with the suggestions of the ESE [21].

Our results demonstrated that the adoption of an appropriate protocol to inactivate viral particles in saliva samples allowed us, during 2021, not only to keep using salivary F as a routine examination but also to reach a higher number than in the pre-COVID-19 period. In the laboratory, saliva samples are processed in batches: the time of heat inactivation does not affect the number of daily processed saliva samples, and the heater for viral inactivation is a facility available in modern laboratories. Therefore, no additional costs, tools, or time were needed: the routine adoption of heat inactivation will not affect negatively the use of salivary cortisol in clinical practice.

In addition, our data indirectly demonstrated that following the aforementioned indications an essential tool for endocrinological activity would have been lost, with potential deleterious consequences for patients with endocrine disease. Our heat inactivation protocol can be applied also to other salivary steroid measurements, after the verification of the non-significant effect of heat in the measured analyte.

Notably, it’s mandatory to specify that no outbreaks of infections among the employers in the Laboratory Medicine (physicians, residents, nurses, laboratory technicians were reported. Therefore, handling salivary specimens could be indirectly considered safe.

Nowadays, thanks to vaccinations and proactive COVID-19 management, Sars-CoV 2 infection will be endemic, with a consistent number of paucisymptomatic new cases. According to all these concepts, the use of salivary F should not be discouraged or avoided.

## Data Availability

All data generated or analyzed during this study are included in this published article or in its supplementary data (reference number 25).
